# Revisiting TNF Receptor-Associated Periodic Syndrome (TRAPS): Current Perspectives

**DOI:** 10.3390/ijms21093263

**Published:** 2020-05-05

**Authors:** Cornelia Cudrici, Natalie Deuitch, Ivona Aksentijevich

**Affiliations:** 1 Translational Vascular Medicine Branch, National Heart, Lung and Blood Institute, National Institutes of Health, Bethesda, MD 20892, USA; cudricicd@mail.nih.gov; 2 Inflammatory Disease Section, National Human Genome Research Institute, National Institutes of Health, Bethesda, MD 20892, USA; natalie.deuitch@nih.gov

**Keywords:** tumor necrosis factor receptor-associated periodic syndrome (TRAPS), TNFR1, AA amyloidosis, autoinflammatory disorders, misfolding disease, TNF inhibitors, IL-1inhibitors

## Abstract

Tumor necrosis factor receptor-associated periodic syndrome (TRAPS) is an autosomal dominant autoinflammatory syndrome characterized by prolonged and recurrent episodes of fever, abdominal and/or chest pain, arthralgia, myalgia, and erythematous rash. TRAPS is associated with heterozygous variants in the *TNFRSF1A* gene, which encodes the TNFR1 (tumor necrosis factor receptor 1) receptor. Disease-causing variants are found exclusively in the extracellular domain of TNFR1 and affect receptor structure and binding to the TNF ligand. The precise mechanism of the disease is still unclear, but it is thought that intracellular accumulation of misfolded mutant protein leads to endoplasmic reticulum stress and enhanced inflammatory responses through constitutive activation of various immune pathways. Other possible mechanisms contributing to the disease pathogenesis include defective receptor shedding, TNF-induced cell death, production of reactive oxygen species, and autophagy impairment. Patients’ leucocytes are hyperresponsive to stimulation and produce elevated levels of proinflammatory cytokines. Systemic autoimmune (AA) amyloidosis is an important cause of morbidity and mortality in TRAPS. Over the last two decades, new therapies have changed the progression and outcome of the disease. In this review, we summarize clinical data from 209 patients with validated pathogenic variants reported in the literature and discuss TRAPS diagnosis, pathogenesis, and treatment options.

## 1. Introduction

Tumor necrosis factor receptor-associated periodic syndrome (TRAPS) was described in the seminal paper that introduced the concept of autoinflammatory diseases—a distinct group of rheumatological disorders caused by dysregulation of the innate immune system [1]. At that time, the only known autoinflammatory diseases were recessively inherited Familial Mediterranean Fever (FMF) and a dominantly inherited TRAPS. Subsequently, more than 30 genes have been linked to Mendelian autoinflammatory diseases. Patients present with recurrent or chronic systemic inflammation that is primarily driven by activated myeloid cells and can affect a number of tissues and organs, including skin, joints/bones, gastrointestinal tract, spleen, liver, and eyes. Most disease-associated genes are highly expressed in hematopoietic cells, however there are several enzyme deficiencies linked to systemic autoinflammatory diseases (also known as SAIDs) and these mutant genes are ubiquitously expressed [2]. The immunological phenotype of SAIDs was initially characterized by an absence of high-titer autoantibodies and activated T lymphocytes, however more recent studies suggest a role for adaptive immune cells in pathogenesis of many SAIDs. Likewise, autoinflammatory signature has been identified in many autoimmune diseases and primary immunodeficiencies [3,4].

TRAPS was initially described in 1982 in a large Irish family with 16 affected members over three generations. They presented with recurrent attacks of fever, myalgia, and skin rashes, along with leukocytosis and elevated sedimentation rate. The disease was named familial Hibernian fever, however, it was suspected at the time that this could be a variant presentation of FMF [5]. Besides the inconsistency regarding the mode of inheritance—FMF is a recessive disease—TRAPS patients have longer lasting recurrent episodes of inflammation and poor response to treatment with colchicine, which is still a mainstay therapy for FMF.

TRAPS is caused by germline heterozygous pathogenic variants in the TNF receptor superfamily member 1A *(TNFRSF1A*) gene on chromosome 12, which encodes the TNF (tumor necrosis factor) Receptor 1 [1]. TRAPS has been reported in multiple ethnic groups from all over the world, which is consistent with dominant inheritance [6].

The disease is characterized by recurrent fever attacks, sometimes lasting for weeks, accompanied by severe abdominal pain, myalgia, arthralgia, migratory erythematous rash, and eye inflammation, but typically the central nervous system (CNS) is spared [7]. During febrile episodes, patients have elevated acute phase reactants and some can have extremely high levels of serum amyloid A (SAA) predisposing them to AA amyloidosis. Before TRAPS was characterized at a molecular level, SAA amyloidosis was a severe consequence of disease and was associated with high morbidity and mortality. Since then, significant progress has been made in therapeutic options from nonsteroidal anti-inflammatory drugs (NSAIDs) and corticosteroids to biological agents. In particular, TNF inhibitors and interleukin-1 (IL-1) inhibitors have been shown to be very effective in controlling the disease activity and in prevention of SAA amyloidosis.

## 2. TRAPS Genetics

TNFR1/p55/CD120 (TNF Receptor Superfamily Member 1A) belongs to a superfamily of TNF receptors that includes 29 proteins in humans, which interact with one or more cytokines of the TNF family. TNFR1, along with Fas/CD95/TNFRSF6, DR3/TNFRSF25 (death receptor 3), TRAIL receptor 1/TNFRSF10A, TRAIL receptor 2/TNFRSF10, TNF-related apoptosis-inducing ligand receptor 1B, DR6/TNFRSF1, and EDAR (ectodermal dysplasia receptor), form a subgroup of the TNFR superfamily that is comprised of a conserved six alpha-helical fold cytoplasmic intracellular domain, termed a “death domain”, which can induce apoptosis. Besides its role in cell death regulation, ligand binding to these receptors leads to formation of a membrane complex with a role to activate the NF-κB inflammatory pathway or caspase-mediated signaling [8].

**Figure 1:**
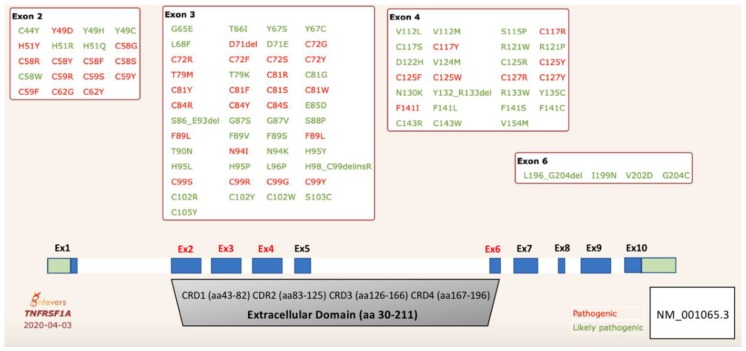
Schematic representation of variants in the *TNFRSF1* gene associated with tumor necrosis factor receptor-associated periodic syndrome (TRAPS). Pathogenic variants are found predominately in the first two cysteine-rich domains, CRD1 and CRD2. The numbering system for TNFR1 (tumor necrosis factor receptor 1) begins at amino acid residue methionine 1. The CRD domains are defined based on the UniProtKB database [9].

The *TNFRSF1* gene is comprised of 10 coding exons (Figure 1). The TNFR1 protein consists of a 29 amino acid N-terminal signal peptide, extracellular domain (residues 30–211), transmembrane (residues 212–232), and a C-terminal cytoplasmic domain (residues 233–455) that includes the death domain (residues 356–441) [9,10]. TNFR1 is a type II transmembrane protein, which can be cleaved from cells by proteolytic processing to form soluble ‘cytokine-like’ molecules [11]. The extracellular domain of TNFR1 has a number of ligand-binding cysteine-rich residues engaged in the formation of highly conserved disulfide bonds, three in each cysteine-rich domains (CRD) [12].

**Figure 2:**
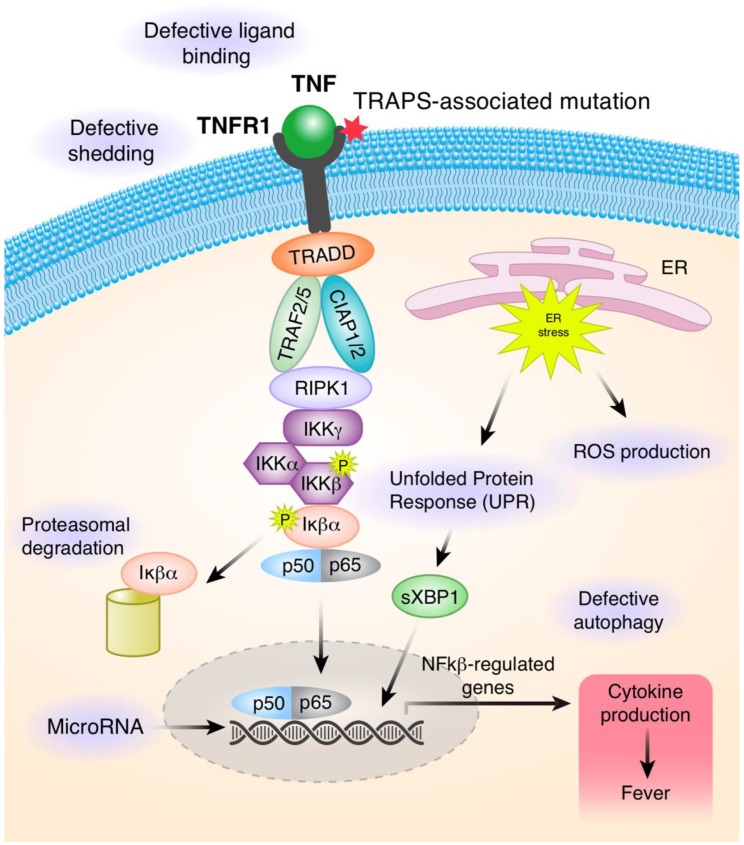
Summary of proposed pathogenic mechanisms in TRAPS. Binding of TNFα to TNFR1 leads to the assembly of the signaling pathway that ultimately upregulates the gene expression of many pro-inflammatory cytokines. There are multiple mechanisms that contribute to the pathogenesis of TRAPS. Heterozygous variants affect the structure of the extracellular domain and impact its ability to bind to the TNF ligand. Mutant receptors fail to shed from the cell surface to generate soluble TNFR1 proteins, which function to attenuate signaling through the TNFR1 receptor. Mutated misfolded proteins accumulate in the cells and cause endoplasmic stress (ER), upregulation in the unfolded protein response (UPR), and increased production of mitochondrial reactive oxygen species (ROS). The UPR initiates ER membrane stress sensors, including inositol-requiring protein (IRE1)α, to restore protein folding and homeostasis in the ER. In the ER stress, activation of IRE1α leads to splicing of transcription factor X-box binding protein 1 (XBP1) into its active form sXBP1, which acts as a transcription factor that can upregulate expression of many target genes. Autophagy is responsible for clearance of intracellular TNFR1. However, in patients with TRAPS, autophagy is defective and mutated proteins are not efficiently cleared from cells. MicroRNA can regulate gene expression at the transcriptional and post-transcriptional levels by binding to the complementary mRNA sequence. MicroRNAs can be detected in serum and various miRNAs can serve as biomarkers of the disease activity.

Binding of TNF to the extracellular domain leads to receptor homotrimerization and formation of the protein complex, referred to as complex I. The aggregated death domains provide interface for interaction with the death domain of TNFR1-associated death domain protein (TRADD). This results in the recruitment of other proteins, including E3 ubiquitin-protein ligase TNF receptor-associated factor 2 and 5 (TRAF2/5), cellular inhibitors of apoptosis 1 and 2 (cIAP1/2), and receptor-interacting serine/threonine-protein kinase 1 (RIPK1). RIPK1 and other components of the complex are rapidly ubiquitinated by cIAP1/2 with Lys (K) 63 Ub-linkage and subsequently, with linear (Met1) Ub-linkage by Linear Ubiquitin Chain Assembly Complex (LUBAC) to promote signaling. This complex activates at least two distinct signaling cascades, NF-kappa-B and cell death patways (Figure 2).

To date, 170 missense sequence variants in the *TNFRSF1A* gene have been described in the gnomAD database. The gene is intolerant to loss-of-function variants (probability of being loss-of-function intolerant, pLI = 0.99) and there are very few frameshift and splice-site variants reported in large population databases. The clinical significance of these variants is unknown. While most missense variants reside in the transmembrane and death domains, TRAPS causal variants are found exclusively in the extracellular domain, encoded by exons 2–6 (Figure 1). The extracellular domain consists of 4 cysteine-rich domains (CRDs) that have a crucial role in protein self-assembly/homotrimerization (CRD1) and ligand binding (CRD2 and 3). Several likely pathogenic variants have been identified in exon 6, which encodes the transmembrane domain, and these variants may affect the receptor cleavage.

To date, 43 missense variants have been validated as pathogenic and 56 as likely pathogenic variants in the *Infevers* database [13,14] (Figure 1). Confirmed TRAPS-associated variants are defined as structural as they disrupt folding of the extracellular domain. Most patients with TRAPS are carriers for missense variants that affect one of the cysteine residues involved in the formation of disulfide bonds in CRD1 and CRD2 [7,15]. In addition, multiple disease-associated missense substitutions have been identified at each of these cysteine residues, confirming their critical function in the folding of the extracellular domain. The most common pathogenic variant p.Thr79Met (T79M, also known as T50M) affects formation of a highly conserved hydrogen bond that is also crucial for protein folding [6]. These structural variants are associated with a more severe phenotype and a higher predisposition to amyloidosis. Initially, it was reported that about 14% of patients with TRAPS developed SAA amyloidosis, however this number is now much lower with better therapy for TRAPS [7]. There are also pathogenic missense variants identified at other amino acids in CRDs, but their effect on secondary structure and protein function is unclear [16]. It should be noted that two numbering systems are used in the literature to define TRAPS causal variants. In the initial report, authors designated the pathogenic variants by subtracting the first 29 amino acids that encode the signal peptide, while, by convention, the numbering should start from the methionine 1 of the official transcript isoform.

Distinct from the pathogenic structural variants in TNFR1 are a few known low-penetrance variants, most notably p.Arg121Glu (R121Q, also known as R92Q) and p.Pro75Leu (P75L, also known as P46L). The P46L variant is exclusively found in people of African ancestry (minor allele frequency, MAF = 0.08), while R92Q is found primarily in Caucasian populations (MAF = 0.02). These variants have been linked to a number of inflammatory diseases in addition to TRAPS, including periodic fever, aphthous stomatitis, pharyngitis, adenitis (PFAPA), multiple sclerosis, and Behcet’s disease. However, at the functional level, these variants behave similarly to TNFR1 wild-type receptors, while structural analysis suggested that they may have an impact on protein folding [15,17,18,19]. Of special interest is the R92Q variant that has been reported in up to 34% of patients from the Eurofever/EUROTRAPS registry. Patients who carry R92Q are less likely to have a positive family history for periodic fever disorder, present with later onset milder manifestations [6] and have a higher rate of spontaneous resolution than patients with structural variants [20,21]. Patients with low-penetrance variants (R121Q, P75L, D41E, V124M, R133Q) are mostly managed with non-steroidal drugs and do not generally require therapy with biologic agents [22,23]. Another low-penetrance variant p.Thr90Ile (T61I) was described in 49% of Japanese patients with SAIDs, however the clinical significance of this variant remains unclear [22]. Recently, there were two reports of somatic variants in patients with adult-onset TRAPS [24,25,26]. Deep sequencing revealed a 24 bp in-frame deletion present in hematopoietic cells, nail, hair, and in sperm, suggesting gonadal mosaicism in one patient [25]. Similar to patients with germline variant, patients with somatic variants respond well to anti-IL1 therapy.

## 3. Pathophysiology in TRAPS

Over the last two decades, multiple molecular mechanisms were proposed to explain cellular pathophysiology involved in TRAPS. One of the initial observations was that patients with TRAPS have low levels of soluble TNFR1 (sTNFR1) and increased membrane bound-TNFR1 (mTNFR1) in activated leukocytes. This led to the “defective shedding hypothesis“ that was proposed by McDermott et al. [1]. Defect in metalloproteinase-induced shedding is likely responsible for low levels of soluble TNFR1 (sTNFR1), which serves to antagonize binding of TNFα to its receptors, therefore more inflammatory cytokine are available to activate the TNFR1 signaling pathway [1] (Figure 2).

A few years later, Huggins et al. demonstrated that not all TRAPS-associated variants lead to abnormal TNFR1 shedding, and that the shedding defect is cell-specific [27]. Based on this hypothesis, etanercept, a biologic TNF inhibitor and TNF decoy receptor, was used in the treatment of TRAPS patients. Etanercept was only partially beneficial in suppressing TRAPS symptoms, suggesting that the shedding hypothesis may be only one of many mechanisms of the disease.

Given that most of pathogenic variants in TRAPS affect folding of the extracellular domain, it was hypothesized that mutated TNFR1 receptors preferentially interact and become constitutively activated, or that these receptors may have an increased affinity for TNF-α cytokine [28], however this hypothesis was not supported by additional studies [29].

Several studies demonstrated that mutant proteins are found mainly in the form of intracellular inclusions. Lobito et al. [17] showed that mutant TNFR1 proteins can self-associate to form aggregates via disulfide links and that they accumulate in the endoplasmic reticulum (ER). This hypothesis was supported by investigations in a murine model of TRAPS with the p.Thr79Met (T50M) pathogenic variant. The peripheral blood neutrophils from knock-in mice show reduced cell surface expression of TNFR1. Mutant receptors do not bind TNF and their capacity to induce spontaneous apoptosis and NF-κB signaling was reduced [17,30] (Figure 2).

Heterozygous TNFR1-mutant mice were hypersensitive to LPS-induced septic shock, whereas homozygous TNFR1-mutant mice resembled TNFR-deficient mice and were resistant to septic shock. Studies in patients’ primary cells, peripheral blood mononuclear cells (PBMCs), were consistent with observations in knock-in mice. Mutant cells showed enhanced activation of mitogen-activated protein kinases (MAPKs) and increased secretion of proinflammatory cytokines upon stimulation with LPS that was dependent on wild type (WT) TNFR1. Thus, both WT and mutant TNFR1 are necessary to potentiate inflammation in TRAPS. These findings establish a mechanism of pathogenesis whereby full expression of the disease phenotype depends on functional cooperation between WT and mutant proteins.

Reactive oxygen species (ROS) have been implicated in many inflammatory diseases, and increased levels were observed in monocytes derived from TRAPS patients and in cells derived from mouse embryonic fibroblasts (MEFs) harboring TRAPS-associated variants (C62Y/C33Y and T79M/T50M). ROS can be generated via Nicotinamide Adenine Dinucleotide Phosphate (NADPH) oxidases (NOX1–5) and mitochondrial respiratory chain complexes and can have different roles. First, they can have an antimicrobial role in host defense and can also decrease sterile inflammation for the first category. Additionally, mitochondrially generated ROS (mROS) can support antiviral host defense by increasing sterile inflammation [31,32]. The exact mechanism for an increase in mitochondrial ROS in TRAPS mutant cells has not yet been elucidated but is likely related to ER stress signaling activation, secondary to the accumulation of mutated TNFR1 proteins. Bulua et al. showed elevated baseline mitochondrial ROS in both mouse MEFs and human primary cells harboring TRAPS-associated variants. TNFR1 mutant cells exhibit altered mitochondrial function and produce pro-inflammatory cytokines in a mROS-dependent manner (Figure 2). Pharmacological blockade of mROS was efficient in reducing inflammatory cytokine production in cells from TRAPS patients [32].

Accumulation of misfolded protein can activate autophagy and UPR (unfolded protein response) via ER stress signaling pathways. UPR is a highly coordinated response that regulates the expression of multiple genes to reestablish cellular homeostasis or induce apoptosis [33]. Three different UPR signaling pathways are present in eukaryotic cells that include activation of three different ER transmembrane proteins: activating transcription factor 6 (ATF6), inositol-requiring transmembrane kinase/endonuclease 1 (IRE1α and β), and pancreatic ER kinase (PERK). Under ER stress conditions, IRE1α activates several transcription factors, including splicing of X-box binding protein 1 (sXBP1), which then regulates genes involved in ER expansion, protein production, and degradation [34,35] (Figure 2).

In PBMCs from TRAPS patients, high levels of spliced XBP1, elevated PERK, and p-PERK levels have been reported compared with cells derived from healthy donors; however, evidence of classical UPR activation was not found. LPS treatment increases sXBP1 levels in TRAPS patients but not in healthy donors, which was abolished by antioxidant treatment [36]. Because TLR-activated XBP1 was required for the production of proinflammatory cytokines (IL-6 and IL-1) [37], the authors concluded that mROS contributes to enhanced LPS response in mutant cells by promoting sXBP1 signaling [36].

Defective autophagy was also reported in TRAPS monocytes and cell lines carrying the structural TRAPS variants by Bachetti et al. [38]. Treatment with geldanamycin (GA), an activator of autophagy, was shown to rescue membrane localization of mutant TNFR1, to decrease ER accumulation of mutated TNFR1, and subsequently, to reduce inflammatory signature in mutant cells. These effects were reversed by the autophagy inhibitor 3-Methyladenine (3-MA). In contrast, proteasome inhibition by lactacystin did not have any effect, suggesting that proteasome is not a main cellular mechanism for the clearance of TNFR1 proteins. Thus, autophagy may play an important role in mechanisms of TRAPS inflammation, similar to other diseases such as Alzheimer’s or Parkinson disease [38,39].

Innate immune activation via Toll-like receptors (TLRs) was also investigated in TRAPS patients. Stimulation of patient PBMCs with a low dose of lipopolysaccharide (LPS) and TLR4 activator can increase the levels of pro-inflammatory cytokine IL-1β, IL-6, and TNFα. This correlates with clinical observations in TRAPS patients who have exaggerated responses to trivial infections [19]. PBMCs from TRAPS patients with the p.Gly87Val (G87V) mutation were hyper-reponsive to TLR2 and TLR4 stimulations with increased production of IL-8 and granulocyte-macrophage colony-stimulating factor (GM-CSF), indicating that these cytokines are contributing to the TRAPS pathogenesis [40]. In addition, TLR-9 activation of PBMCs from a patient carrying the p.C62Y (C33Y) mutation triggered production of many pro-inflammatory cytokines and activated several inflammatory pathways, including NF-κB (nuclear factor kappa-light-chain-enhancer of activated B cells), JNK (c-Jun N-terminal kinase), and P38 MAPK. Collectively, these data demonstrate hyperactivity of the innate immune reponse in TRAPS patients [41].

Increased levels of proinflammatory cytokine including IL-1β, TNFα, and IL-6 were reported [19,32] secondary to enhanced NF-κB and MAPK signaling activation. IL-1β is an essential cytokine of local and systemic inflammation that is produced mainly by myeloid cells. The observation that therapy with IL-1β antagonists results in a rapid and sustained reduction in disease severity of many autoinflammatory disease including TRAPS indicates that these diseases are predominately mediated by the IL-1 cytokine family [42].

Gene expression studies by microarray identified constitutive overexpression of genes involved in IL1β signaling and redox regulation in TRAPS patients’ monocytes compared to controls. LPS stimulation leads to differential regulation of genes involved in post-translational modifications, protein folding, and ubiquitination, while LPS failed to upregulate interferon type I and II responses [43]. Another gene expression study compared transcriptome of 20 patients with TRAPS and 20 healthy controls before and after initiation of the treatment with canakinumab (a human anti-IL-1β monoclonal antibody). Prior to treatment, TRAPS patients had higher gene expression of *TNFRSF1A*, *IL-1β*, *MAPK14*, and *NFΚB1* compared with healthy donors; however, 15 days after canakinumab treatment, gene expression was completely normalized. This suggests that IL-1β plays an essential role in the TRAPS pathogenesis [44].

MicroRNAs (miRNAs) are small non-coding single-stranded RNA molecules (16 to 27 nucleotides in length) that can regulate gene silencing at the transcriptional and post-transcriptional levels by binding to the complementary mRNA sequence. A single miRNA molecule can target multiples mRNAs and regulate the expression of many protein-coding genes. MicroRNAs can be detected in serum and various miRNAs have been reported as biomarkers in multiple human diseases [45,46,47]. Decreased expression levels of six different circulating miRNAs (miR-134, miR-17-5p, miR-498, miR-451a, miR-572, miR-92a-3p) were found in samples from 15 TRAPS patients compared to controls. Conversely, four miRNAs (miR-150-3p, miR-92a-3p, miR-22-3p, miR-30d-5p) were upregulated in patients’ serum samples following anakinra treatment. In addition, two other miRNAs correlated with TRAPS clinical features: reduced miR-92b levels correlated with an increasing number of TRAPS exacerbation/year, and high levels of miR-377-5p was correlated with an increase in serum amyloid A levels [48]. Harrison et al. reported reduced levels of two different miRNAs (miR-146a and miR-155) in the TRAPS-derived dermal fibroblasts following LPS stimulation compared to control fibroblasts. Upregulation of autophagy via IRE1 arm was associated with decreased levels of miR-146a and miR-155, leading to LPS hyperresponsiveness in TRAPS patients [49].

The high levels of adipokines in TRAPS patients were also reported, but their role in the pathogenesis of disease progression is poorly defined. Adipokines are bioactive molecules mainly produced or released by adipose tissue that acts as paracrine and endocrine hormones and are important regulators of several key processes, including appetite, fat distribution, blood pressure, hemostasis endothelial function, and inflammation [50]. Cantarini et al. [51] evaluated the levels of four adipokines (leptin, resistin, visfatin, and adiponectin) in serum obtained from 14 TRAPS patients carrying cysteine variants, 16 TRAPS patients with non-cysteine variants, and 16 healthy controls. They reported decreased levels of visfatin in patients with cysteine variants compared with the other two groups. Serum leptin was significantly correlated with the number of TRAPS attacks, and serum adiponectin levels were increased in TRAPS patients with amyloidosis. No correlation was found between the levels of the serum adipokines and various treatment regiments (biological or steroid medication) in these patients. Why the adipokine production is different in TRAPS patients with cysteine pathogenic variants compared with TRAPS patients with other variants and healthy control is unclear. Visfatin levels were reported to be downregulated by the TNF-α in vivo [52], while leptin levels can induce activation of monocyte in macrophages, neutrophils, and natural killer cells [53].

The role of adaptive immunity in the pathogenesis of TRAPS was explored by Pucino et al. [54]. Effect of structural variants (cysteine residues) versus low penetrance (non-structural variants) were analyzed on different T cell subsets from 20 patients with highly penetrant variants (HP), 15 patients with low-penetrant (LP) variants, and 32 age- and gender-matched healthy controls. TRAPS-HP patients had a higher conversion of naïve T cells into memory T cells (CD4^+^CD45RO^+^) with an effector phenotype (CD4^+^CD25^+^), similar to patients with classical autoimmune diseases. The conventional CD4 T cells (CD4^+^CD25^−^) from TRAPS-HP patients had a higher proliferation and increased activation of mTOR, ERK1/2, STAT, and NF-κB p65 signaling pathways. TRAPS-HP peripheral Tregs (CD4^+^Fox3^+^) were reduced in number and had a decreased suppressive capacity when compared with Tregs from healthy controls, features that are observed in many autoimmune disorders [15,17,19,36,54,55,56]. In summary, these data show a role for the cells of the adaptive immunity in the pathogenesis of TRAPS.

## 4. Clinical and Laboratory Features in TRAPS Patients

TRAPS is one of the most common inherited recurrent periodic fever syndromes and has an estimated prevalence of one per one million individuals worldwide. Most cases are reported in Caucasian and Asian populations.

The symptoms of TRAPS are heterogeneous and are most likely linked to genotypes, although variable disease penetrance has been noted. Disease onset typically occurs in early childhood (median 4.3 years). However, adult onset of TRAPS (over 30 years old) has been reported in about 10% of the patients, mostly with milder variants [56,57,58,59,60]. Patients with somatic variants also present with later-onset symptoms.

**Figure 3:**
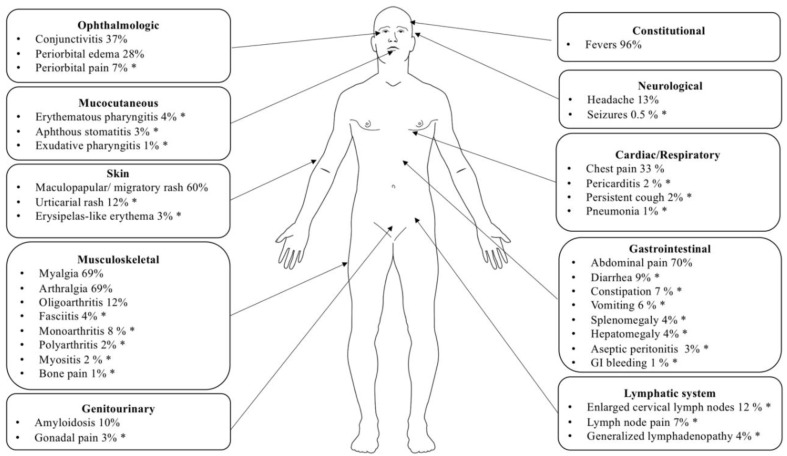
Summary of most common clinical manifestations in TRAPS. Organ-specific clinical manifestation of 209 TRAPS patients with variants validated as pathogenic and likely pathogenic in the *Infevers* database. * Not all reports contained an explicit description of these symptoms and they may be present in a larger proportion of patients than reported here [6,22,57,61,62,63].

Typically, recurrent febrile episodes occur every 4–6 weeks and last from 5 days to 3 weeks. These episodes can be precipitated by emotional and physical stress, minor infections, trauma, hormonal changes, fatigue, vaccinations, but most of the time, the triggering factors remain unknown. Searching the literature, we identified 16 articles that included detailed clinical descriptions of TRAPS patients carrying variants that have been validated in *Infevers* as pathogenic or likely pathogenic. Reports of the low-penetrance R92Q or P46L variants were not included in this analysis. We further selected manuscripts that reported more than four TRAPS cases and excluded duplicate reports of the same patient. We reviewed patients from the following cohorts: 40 patients by Hull et al. [57], 97 patients from the Eurofever/EUROTRAPS international registry by Lachmann et al. [6], 44 patients from the Japanese cohort by Ueda et al. [22], 14 patients from the Greek cohort by Nezos et al. [61], 8 patients from the Chinese cohort by Zhao et al. [62], and 6 patients from the Brazilian cohort by Jesus et al. [63]. In total, 209 patients were analyzed and their 37 clinical features are summarized in Figure 3.

The most common clinical manifestations include fevers (96%), myalgias (69%), arthralgia (69%) erythematous rash (60%), abdominal pain (70%), acute conjunctivitis (37%), periorbital edema (28%), arthritis (22%), lymphadenopathy (16%), hepato or splenomegaly (4%), chest pain (33%), and headache (13%) (Figure 3). Serosal inflammation secondary to TRAPS inflammatory attacks can cause recurrent episodes of pericarditis, peritonitis, pleurisy, and testicular inflammation [64,65,66].

Cutaneous manifestations can be present in up to 75% of patients during an attack. They can consist of migratory erythema, which is centrifugal in distribution, and edematous plaques, in some cases, urticarial rashes or erysipelas-like rashes (Figure 3). Skin biopsy reveals dermal monocytic and lymphocytic infiltrates and immunohistochemically, the infiltrate consists of T cells (CD3+, CD4+, CD8+) and monocytes (CD68+), but are negative for B cells (CD79a− and CD20−), multinucleated macrophages, granulomatous, or leukocytoclastic vasculitis [67,68].

During times of active disease, symptoms are almost always associated with an increase in acute phase reactants: C-reactive protein and erythro-sedimentation rate, serum amyloid A, fibrinogen, haptoglobin and neutrophil leukocytosis, thrombocytosis, and normochromic anemia (secondary to chronic inflammation). Between attacks, acute phase reactants can be elevated, but at lower levels than during a flare. Periodic evaluation of kidney function and urinalysis for the occurrence of proteinuria can be a useful parameter in evaluating clinical activity and response to treatment [69].

AA amyloidosis is the most severe complication of untreated TRAPS patients and is associated with increased morbidity and mortality. Proteinuria and kidney failure occur in 80–90% of cases with amyloidosis, while thyroid, myocardium, liver, and spleen deposits are less common. Patients with pathogenic variants impacting cysteine residues or carrying the p.T79M (T50M) variant are at a higher risk for developing amyloidosis, possibly secondary to longer duration of the flares, and more severe inflammation. High levels of serum AA protein were shown be indicative of amyloid deposition. The prognosis of SAA amyloidosis is linked to the disease activity, and requires effective control of inflammatory attacks [69].

## 5. TRAPS Diagnosis

Over the last two decades, the diagnosis of TRAPS has evolved through the genetic testing and development of new diagnostic criteria for screening and assessment of disease activity. The diagnosis of TRAPS is generally made through a combination of clinical symptoms and laboratory findings during flares and is supported by genetic testing. 

In 2002, Hull et al. evaluated more than 50 TRAPS patients with specific genetic variants [57] and suggested that the diagnosis of TRAPS can be made if the following criteria are met: (1) periodical inflammatory symptoms lasting for over 5 days (fever, rashes, myalgia, abdominal pain, ocular involvement), (2) improvement of the symptoms after treatment with corticosteroids but not with colchicine, and (3) a positive family history [57]. 

In 2011, Piram and colleagues developed an autoinflammatory disease activity index (AIDAI) using patients’ questionnaires (from 4 TRAPS patients) and scored by physicians [70]. The activity score was based on the patients’ symptoms, and along with the fever, the most characteristic manifestations included abdominal pain, localized myositis, skin erythema, and periorbital edema. This study was validated three years later in 14 TRAPS patients (6 patients with inactive disease, 3 with severe activity, 3 with mild activity, and 2 with low activity). The AIDAI score includes fever, overall well-being (described by patients), abdominal pain, nausea/vomiting, diarrhea, headache, chest pain, painful subcutaneous nodules, arthralgia/myalgia, swelling of the joints, eye manifestation, and skin rashes, with each item dichotomides as yes or no. A score over 9 indicates active disease and a score of less than 9 identifies patients as having inactive disease. 

Using data from the Eurofever/EUROTRAPS international registry that included 158 TRAPS and TRAPS-like patients with molecular diagnosis, Lachmann et al. reported the main symptoms as being: fever (88%), limb pain (85%), abdominal pain (74%), and skin rashes (63%). About one third of the cases were pediatric patients, and they had a higher incidence of cervical lymphadenopathy during the attacks (31% versus 9% in adults) [6].

Another set of classification criteria was proposed in 2015 by Federici et al. using clinical data from 86 patients with TRAPS derived from the Eurofever registry. In this study, univariate and multivariate analyses identified the presence of five clinical variables during flares with a specific score: periorbital edema (21 points), duration of episodes over 6 days (19 points), migratory rash (18 points), myalgia (6 points), presence of affected relatives (7 points), absence of vomiting (14 points), and absence of aphthous stomatitis (15 points). The combined score over 43 points had a sensitivity of 85% and a specificity of 87% in the evaluated cohort and 80% sensitivity and 91% specificity in an independent validation cohort of patients. This set of criteria can be used as an indication for genetic testing or as classification criteria to differentiate patients with TRAPS from other autoinflammatory diseases [71].

In 2019, Gattorno et al. reported a new evidence-based classification criteria for TRAPS that was developed and validated by a panel of 33 international expert clinicians and geneticists [72]. They independently and blindly assessed clinical, laboratory, and genetic characteristics of 60 patients until consensus was achieved for the best set of classification criteria. The performance of this final set of classification criteria was then cross-validated in an independent set of a randomly selected 1018 patients from this web registry. The new criteria for TRAPS included: presence of confirmatory *TNFRSF1A* genotype and at least one of the following features: duration of the episode more than 7 days/myalgia/migratory rash/periorbital edema/relatives affected. In the absence of a confirmatory *TNFRSF1A* genotype, at least two among the following clinical manifestations of the disease had to be met: duration of the episode lasting more than 7 days/myalgia/migratory rash/periorbital edema/relatives affected. These new classification criteria include, for the first time, genotype along with clinical features and as such, this combination outperforms all previously reported classification criteria with sensitivity of 95%, specificity of 99%, and accuracy of 99% [72].

For patients where genetic testing is not available, the Eurofever panelists recommend a second set of criteria, with the presence of several clinical variables, both in positive or negative association, and a score higher than 5 had a sensitivity of 87%, a specificity of 92%, and accuracy of 96%. Presence of the following clinical features will contribute points: fever ≥ 7 days (2 points), fever 5–6 days (1 point), migratory rash (1 point), periorbital edema (1 point), myalgia (1 point), and positive family history (1 point). Absence of the aphthous stomatitis and pharyngotonsillitis will add one point for each. Validation of the new classification criteria was performed in an independent set of molecularly confirmed 116 TRAPS patients with good sensitivity and specificity (74% and 100%) [72].

## 6. TRAPS Treatment

Prior to identification of the genetic etiology of TRAPS, patients were treated with multiple medications including colchicine, methotrexate, cyclosporine, or thalidomide, with minimal benefits. High doses of corticosteroids are generally effective in reducing inflammation; however, prolonged treatment and escalating doses are required to control chronic TRAPS symptoms. Long-term therapy with corticosteroids was not effective in preventing the development of amyloidosis or in reducing frequency or intensity of inflammatory attacks [57].

TNF-α blocking agents were introduced based on the observation that soluble p55 TNFR1 was found to be decreased in the serum of TRAPS patients. The first TNF-α inhibitor used was etanercept, a fusion protein combining two molecules of the human TNF-R2 (p75) with an IgG1 Fc fragment. Etanercept has shown efficacy and safety in several case series [73,74]. In an open-label study of patients with TRAPS, a significant attenuation of symptoms, and decrease in frequency and duration of attacks were observed in a dose-dependent manner, but without complete resolution of symptoms. Etanercept also reduced the levels of acute-phase reactants during asymptomatic periods and decreased the use of NSAIDs and glucocorticoids [57,75,76,77]. Treatment with etanercept might be efficacious in preventing AA-amyloidosis in some patients [76,77,78]. Based on data from the Eurofever registry, Haar and collaborators showed that etanercept was beneficial in more than 80% of TRAPS patients; however, only 30% of patients achieved a complete response [79].

In contrast, other TNF-α inhibitors: adalimumab (a full humanized anti-TNF monoclonal antibody) and infliximab (a chimeric mouse-human monoclonal antibody) were reported to cause paradoxical worsening of disease flare in TRAPS patients. The following mechanisms have been proposed to this effect: treatment with infliximab prevents caspase-3-induced apoptotic activity in TRAPS PBMCs and may lead to c-Rel-mediated increase in secretion of proinflammatory cytokines (IL-1β, IL-6, IL-8, and IL-12). A difference in binding activity to TNF and in drug kinetics may also explain the differential effect of TNF inhibitors on mutant primary cells [80,81,82]. One study looked at a variance in binding affinities to soluble TNF-α and complement-activating properties among these TNF antagonists, however, results could not explain difference in their clinical efficacy [81].

Treatment with IL-6 inhibitor tocilizumab (a humanized monoclonal antibody that competitively inhibits the binding of IL-6 to its receptor) was reported in patients who did not respond to TNF or IL-1 inhibitors [83,84]. Several case reports were published in patients with various TNFR1 variants: one patient with R92Q, the second patient with no identified variant, the third patient with C33Y, and the fourth patient with C96R, were all treated with tocilizumab with complete resolution of their symptoms [83,84,85,86].

Similar to other IL-1 β-mediated autoinflammatory diseases, known as inflammasomopathies, including familial Mediterranean fever (FMF), cryopyrin-associated periodic syndrome (CAPS), and mevalonate kinase deficiency (MKD), anti-IL agents represent an important therapeutic option for TRAPS patients [87]. Initially, IL-1 inhibitors were used in TRAPS patients refractory to etanercept medication [88]. Anakinra, a recombinant non-glycosylated human interleukin-1 receptor antagonist (IL-1Ra), has been shown to be efficient in controlling clinical manifestations in TRAPS, normalization of inflammatory markers, and in prevention of disease relapse and complications [89,90]. Several studies reported a dramatic resolution of TRAPS symptoms after anakinra administration, even when administered on-demand [91,92].

The second IL-1 inhibitor, canakinumab, a human anti-IL-1β monoclonal antibody, which is FDA-approved for the treatment of multiple autoinflammatory diseases (including: CAPS, MKD/Hyperimmunoglobulin D Syndrome and in colchicine-resistant FMF), was approved by the FDA for TRAPS in 2013. TRAPS patients were successfully treated with canakinumab as demonstrated by complete remissions of all the clinical symptoms and normalization of their inflammatory markers [86,93,94]. Gattorno et al. evaluated the efficacy of canakinumab in 20 TRAPS patients in a phase II clinical trial over a 4 month period, followed by a 5 month withdrawal period and with reintroduction of canakinumab on disease flare for long-term treatment for another 24 months. The primary endpoint was clinical remission at day 15 and full or partial serological remission. Most of the patients (95%) achieved primary endpoint, and after the canakinumab was withdrawn, all the patients relapsed with a median time to relapse of 91.5 days. After the reintroduction of canakinumab, all the patients underwent clinical and serological remission, indicating that clinical benefits are achieved during long-term treatment [95]. A phase III randomized, double-blind placebo-controlled study was published in 2016: 46 TRAPS patients were followed for a 12 week screening period and then randomized at the flare onset to a 16 week treatment period with 150 mg every four weeks for canakinumab or placebo. The primary outcome was the resolution of the index flare by day 15 and no subsequent flares for up to a week. The proportion of responders at the end of week 16 was 45% (10/22 patients) in the treatment group and 8% (2/24 patients) in the placebo group. During the withdrawal period, where the canakinumab was administered over a prolonged dosing interval (8 weeks), 53% of the patients maintained controlled disease [96]. Data from multiple clinical studies confirm the efficacy and safety of biological medications in controlling TRAPS symptoms and a long-term benefit for these patients. Adverse reactions are mild, and consist of mild infections, injection site reactions, and in general, these drugs are well-tolerated by patients. Taken together, biological strategies that block specific immune mediators such us TNF-α or IL-1 β are effective in suppressing TRAPS symptoms, preventing reactive amyloidosis, and halting the progression to organ damage. Table 1 summarizes the main studies conducted in TRAPS patients on various biological medications.

## 7. Conclusions

TRAPS is one of the more common monogenic autoinflammatory diseases that affects patients worldwide. Despite significant advances in the characterization of genetic causes of TRAPS, the molecular pathophysiology of this disease remains to be elucidated. It is essential for clinicians to recognize the clinical features of TRAPS patients. Childhood-onset long-lasting recurrent fevers with severe abdominal pain, myalgia/arthralgia, rash, and a positive family history are indicative of TRAPS, and all patients suspected to have TRAPS should be evaluated by genetic testing for germline variants in *TNFRSF1A*. Classification criteria that include genotype outperform classification criteria based on clinical features by sensitivity and specificity. TRAPS patients with structural variants and high CRPs should be treated with biological agents (IL-1 or IL-6 blockers, etanercept) and screened regularly for proteinuria. More studies are necessary to evaluate the long-term effects of these medications on TRAPS symptoms and its complications. Taken together, biological strategies that block specific immune mediators such us TNF-α or IL-1β are effective in suppressing the disease activity, preventing reactive amyloidosis, and halting the progression of organ damage.

## Figures and Tables

**Table 1:** Summay of studies that have investigated treatment of TRAPS with biological therapies: TNF inibitors, IL-1 inhibitors and Il-6 inhibitors.

Biological Medication Used	Type of Study	Dose of Medication	Follow-up Period	Number of Patients	Adverse Events	Ref.
Etanercept	Case report	0.4 mg/kg 2× week	12 months	1	minor injection site reactions	[73]
	Open label study	NA	24 weeks	7	NA	[74]
	Open label study	25 mg 2× week	18 months	3	NA	[77]
	Open label study	25 mg 2× week	24 weeks	7	minor injection site reactions, upper respiratory tract infections	[76]
	Open label study	25 mg (adult dose) or 0.4 mg/kg (pediatric dose) 2× week	6 months	9	NA	[57]
	Open-label dose escalation study	25 mg 2× week for 14 weeks, then 25 mg 3× week for 14 weeks	10 years	15	Injection site reactions	[75]
Anakinra	Case report	100 mg daily	3 months	1	Injection site reactions	[88]
	Open-label dose	100 mg daily	On demand	2	Injection site reactions	[92]
	Open-label dose	100 mg daily	12–46 months	7	Injection site reactions, respiratory and urinary infections	[91]
	Open-label dose	1.5 mg/kg daily	15 days	4	Injection site reactions	[95]
Canakinumab	Case report	150 mg every 8 weeks	18 months	1	NA	[93]
	Case report	150 mg every 4 weeks	47 months	1	NA	[94]
	Case report	4 mg/kg mg every 8 weeks	36 months	1	NA	[86]
	Phase II, open-label study 2b	150 mg every 4 weeks (2 mg/kg pediatric patients)	33 months	20	Nasopharingitis, abdominal pain, headache	[95]
	Phase III, randomized Double-blind, placebo-controlled study	150 mg every 4 weeks (2 mg/kg if weight less 40 kg)	4 months	22	Nasopharingitis, injection site reactions, upper respiratory tract infection	[96]
	Phase III, Randomized, withdrawal period study	150 mg every 8 weeks (2 mg/kg if weight less 40 kg)	6 months	4	Nasopharingitis, injection site reactions, upper respiratory tract infection	[96]
Tocilizumab	Case report	8 mg/kg every 4 weeks	6 months	1	NA	[83]
	Case report	8 mg/kg every 2 weeks then PRN	>6 months	1	NA	[84]
	Case report	8 mg/kg every 4 weeks	6 months	1	Trombocytopenia	[85]
	Case report	8 mg/kg every 4 weeks	42 months	1	NA	[86]

NA, non applicable.

## References

[1] McDermott M.F., Aksentijevich I., Galon J., McDermott E.M., Ogunkolade B.W., Centola M., Mansfield E., Gadina M., Karenko L., Pettersson T. (1999). Germline mutations in the extracellular domains of the 55 kDa TNF receptor, TNFR1, define a family of dominantly inherited autoinflammatory syndromes. Cell.

[2] Beck D.B., Aksentijevich I. (2019). Biochemistry of Autoinflammatory Diseases: Catalyzing Monogenic Disease. Front. Immunol..

[3] Savic S., Caseley E.A., McDermott M.F. (2020). Moving towards a systems-based classification of innate immune-mediated diseases. Nat. Rev. Rheumatol..

[4] Manthiram K., Zhou Q., Aksentijevich I., Kastner D.L. (2017). The monogenic autoinflammatory diseases define new pathways in human innate immunity and inflammation. Nat. Immunol..

[5] Williamson L.M., Hull D., Mehta R., Reeves W.G., Robinson B.H., Toghill P.J. (1982). Familial Hibernian fever. QJM Int. J. Med..

[6] Lachmann H.J., Papa R., Gerhold K., Obici L., Touitou I., Cantarini L., Frenkel J., Anton J., Kone-Paut I., Cattalini M. (2014). The phenotype of TNF receptor-associated autoinflammatory syndrome (TRAPS) at presentation: A series of 158 cases from the Eurofever/EUROTRAPS international registry. Ann. Rheum. Dis..

[7] Aksentijevich I., Galon J., Soares M., Mansfield E., Hull K., Oh H.H., Goldbach-Mansky R., Dean J., Athreya B., Reginato A.J. (2001). The tumor-necrosis-factor receptor-associated periodic syndrome: New mutations in TNFRSF1A, ancestral origins, genotype-phenotype studies, and evidence for further genetic heterogeneity of periodic fevers. Am. J. Hum. Genet..

[8] Croft M., Siegel R.M. (2017). Beyond TNF: TNF superfamily cytokines as targets for the treatment of rheumatic diseases. Nat. Rev. Rheumatol..

[9] UniProt C. (2019). UniProt: A worldwide hub of protein knowledge. Nucleic Acids Res..

[10] Banner D.W., D’Arcy A., Janes W., Gentz R., Schoenfeld H.J., Broger C., Loetscher H., Lesslauer W. (1993). Crystal structure of the soluble human 55 kd TNF receptor-human TNF beta complex: Implications for TNF receptor activation. Cell.

[11] Cui X., Hawari F., Alsaaty S., Lawrence M., Combs C.A., Geng W., Rouhani F.N., Miskinis D., Levine S.J. (2002). Identification of ARTS-1 as a novel TNFR1-binding protein that promotes TNFR1 ectodomain shedding. J. Clin. Investig..

[12] Bodmer J.L., Schneider P., Tschopp J. (2002). The molecular architecture of the TNF superfamily. Trends Biochem. Sci..

[13] Sarrauste de Menthiere C., Terriere S., Pugnere D., Ruiz M., Demaille J., Touitou I. (2003). INFEVERS: The Registry for FMF and hereditary inflammatory disorders mutations. Nucleic Acids Res..

[14] Van Gijn M.E., Ceccherini I., Shinar Y., Carbo E.C., Slofstra M., Arostegui J.I., Sarrabay G., Rowczenio D., Omoyimni E., Balci-Peynircioglu B. (2018). New workflow for classification of genetic variants’ pathogenicity applied to hereditary recurrent fevers by the International Study Group for Systemic Autoinflammatory Diseases (INSAID). J. Med. Genet..

[15] Rebelo S.L., Bainbridge S.E., Amel-Kashipaz M.R., Radford P.M., Powell R.J., Todd I., Tighe P.J. (2006). Modeling of tumor necrosis factor receptor superfamily 1A mutants associated with tumor necrosis factor receptor-associated periodic syndrome indicates misfolding consistent with abnormal function. Arthritis Rheum..

[16] Churchman S.M., Church L.D., Savic S., Coulthard L.R., Hayward B., Nedjai B., Turner M.D., Mathews R.J., Baguley E., Hitman G.A. (2008). A novel TNFRSF1A splice mutation associated with increased nuclear factor kappaB (NF-kappaB) transcription factor activation in patients with tumour necrosis factor receptor associated periodic syndrome (TRAPS). Ann. Rheum. Dis..

[17] Lobito A.A., Kimberley F.C., Muppidi J.R., Komarow H., Jackson A.J., Hull K.M., Kastner D.L., Screaton G.R., Siegel R.M. (2006). Abnormal disulfide-linked oligomerization results in ER retention and altered signaling by TNFR1 mutants in TNFR1-associated periodic fever syndrome (TRAPS). Blood.

[18] Ravet N., Rouaghe S., Dode C., Bienvenu J., Stirnemann J., Levy P., Delpech M., Grateau G. (2006). Clinical significance of P46L and R92Q substitutions in the tumour necrosis factor superfamily 1A gene. Ann. Rheum. Dis..

[19] Simon A., Park H., Maddipati R., Lobito A.A., Bulua A.C., Jackson A.J., Chae J.J., Ettinger R., de Koning H.D., Cruz A.C. (2010). Concerted action of wild-type and mutant TNF receptors enhances inflammation in TNF receptor 1-associated periodic fever syndrome. Proc. Natl. Acad. Sci. USA.

[20] Pelagatti M.A., Meini A., Caorsi R., Cattalini M., Federici S., Zulian F., Calcagno G., Tommasini A., Bossi G., Sormani M.P. (2011). Long-term clinical profile of children with the low-penetrance R92Q mutation of the TNFRSF1A gene. Arthritis Rheum..

[21] Ruiz-Ortiz E., Iglesias E., Soriano A., Bujan-Rivas S., Espanol-Rego M., Castellanos-Moreira R., Tome A., Yague J., Anton J., Hernandez-Rodriguez J. (2017). Disease Phenotype and Outcome Depending on the Age at Disease Onset in Patients Carrying the R92Q Low-Penetrance Variant in TNFRSF1A Gene. Front. Immunol..

[22] Ueda N., Ida H., Washio M., Miyahara H., Tokunaga S., Tanaka F., Takahashi H., Kusuhara K., Ohmura K., Nakayama M. (2016). Clinical and Genetic Features of Patients With TNFRSF1A Variants in Japan: Findings of a Nationwide Survey. Arthritis Rheumatol..

[23] Cantarini L., Rigante D., Merlini G., Vitale A., Caso F., Lucherini O.M., Sfriso P., Frediani B., Punzi L., Galeazzi M. (2014). The expanding spectrum of low-penetrance TNFRSF1A gene variants in adults presenting with recurrent inflammatory attacks: Clinical manifestations and long-term follow-up. Semin. Arthritis Rheum..

[24] Hoffman H.M., Broderick L. (2017). Editorial: It Just Takes One: Somatic Mosaicism in Autoinflammatory Disease. Arthritis Rheumatol..

[25] Rowczenio D.M., Trojer H., Omoyinmi E., Arostegui J.I., Arakelov G., Mensa-Vilaro A., Baginska A., Silva Pilorz C., Wang G., Lane T. (2016). Brief Report: Association of Tumor Necrosis Factor Receptor-Associated Periodic Syndrome With Gonosomal Mosaicism of a Novel 24-Nucleotide TNFRSF1A Deletion. Arthritis Rheumatol..

[26] Kontzias A., Zarabi S.K., Calabrese C., Wang Y., Judis L., Yao Q., Cheng Y.W. (2019). Somatic mosaicism in adult-onset TNF receptor-associated periodic syndrome (TRAPS). Mol. Genet. Genom. Med..

[27] Huggins M.L., Radford P.M., McIntosh R.S., Bainbridge S.E., Dickinson P., Draper-Morgan K.A., Tighe P.J., Powell R.J., Todd I. (2004). Shedding of mutant tumor necrosis factor receptor superfamily 1A associated with tumor necrosis factor receptor-associated periodic syndrome: Differences between cell types. Arthritis Rheum..

[28] Yousaf N., Gould D.J., Aganna E., Hammond L., Mirakian R.M., Turner M.D., Hitman G.A., McDermott M.F., Chernajovsky Y. (2005). Tumor necrosis factor receptor I from patients with tumor necrosis factor receptor-associated periodic syndrome interacts with wild-type tumor necrosis factor receptor I and induces ligand-independent NF-kappaB activation. Arthritis Rheum..

[29] Kimberley F.C., Lobito A.A., Siegel R.M., Screaton G.R. (2007). Falling into TRAPS--receptor misfolding in the TNF receptor 1-associated periodic fever syndrome. Arthritis Res..

[30] Yang Q., Kim Y.S., Lin Y., Lewis J., Neckers L., Liu Z.G. (2006). Tumour necrosis factor receptor 1 mediates endoplasmic reticulum stress-induced activation of the MAP kinase JNK. EMBO Rep..

[31] Pelletier M., Lepow T.S., Billingham L.K., Murphy M.P., Siegel R.M. (2012). New tricks from an old dog: Mitochondrial redox signaling in cellular inflammation. Semin. Immunol..

[32] Bulua A.C., Simon A., Maddipati R., Pelletier M., Park H., Kim K.Y., Sack M.N., Kastner D.L., Siegel R.M. (2011). Mitochondrial reactive oxygen species promote production of proinflammatory cytokines and are elevated in TNFR1-associated periodic syndrome (TRAPS). J. Exp. Med..

[33] Walter P., Ron D. (2011). The unfolded protein response: From stress pathway to homeostatic regulation. Science.

[34] Wu J., Kaufman R.J. (2006). From acute ER stress to physiological roles of the Unfolded Protein Response. Cell Death Differ..

[35] Bravo R., Parra V., Gatica D., Rodriguez A.E., Torrealba N., Paredes F., Wang Z.V., Zorzano A., Hill J.A., Jaimovich E. (2013). Endoplasmic reticulum and the unfolded protein response: Dynamics and metabolic integration. Int. Rev. Cell Mol. Biol..

[36] Dickie L.J., Aziz A.M., Savic S., Lucherini O.M., Cantarini L., Geiler J., Wong C.H., Coughlan R., Lane T., Lachmann H.J. (2012). Involvement of X-box binding protein 1 and reactive oxygen species pathways in the pathogenesis of tumour necrosis factor receptor-associated periodic syndrome. Ann. Rheum. Dis..

[37] Martinon F., Chen X., Lee A.H., Glimcher L.H. (2010). TLR activation of the transcription factor XBP1 regulates innate immune responses in macrophages. Nat. Immunol..

[38] Bachetti T., Chiesa S., Castagnola P., Bani D., Di Zanni E., Omenetti A., D’Osualdo A., Fraldi A., Ballabio A., Ravazzolo R. (2013). Autophagy contributes to inflammation in patients with TNFR-associated periodic syndrome (TRAPS). Ann. Rheum. Dis..

[39] Nedjai B., Hitman G.A., Yousaf N., Chernajovsky Y., Stjernberg-Salmela S., Pettersson T., Ranki A., Hawkins P.N., Arkwright P.D., McDermott M.F. (2008). Abnormal tumor necrosis factor receptor I cell surface expression and NF-kappaB activation in tumor necrosis factor receptor-associated periodic syndrome. Arthritis Rheum..

[40] Tsuji S., Matsuzaki H., Iseki M., Nagasu A., Hirano H., Ishihara K., Ueda N., Honda Y., Horiuchi T., Nishikomori R. (2019). Functional analysis of a novel G87V TNFRSF1A mutation in patients with TNF receptor-associated periodic syndrome. Clin. Exp. Immunol..

[41] Negm O.H., Singh S., Abduljabbar W., Hamed M.R., Radford P., McDermott E.M., Drewe E., Fairclough L., Todd I., Tighe P.J. (2019). Patients with tumour necrosis factor (TNF) receptor-associated periodic syndrome (TRAPS) are hypersensitive to Toll-like receptor 9 stimulation. Clin. Exp. Immunol..

[42] Dinarello C.A., van der Meer J.W. (2013). Treating inflammation by blocking interleukin-1 in humans. Semin. Immunol..

[43] Borghini S., Ferrera D., Prigione I., Fiore M., Ferraris C., Mirisola V., Amaro A.A., Gueli I., Zammataro L., Gattorno M. (2016). Gene expression profile in TNF receptor-associated periodic syndrome reveals constitutively enhanced pathways and new players in the underlying inflammation. Clin. Exp. Rheumatol..

[44] Torene R., Nirmala N., Obici L., Cattalini M., Tormey V., Caorsi R., Starck-Schwertz S., Letzkus M., Hartmann N., Abrams K. (2017). Canakinumab reverses overexpression of inflammatory response genes in tumour necrosis factor receptor-associated periodic syndrome. Ann. Rheum. Dis..

[45] Bartel D.P. (2004). MicroRNAs: Genomics, biogenesis, mechanism, and function. Cell.

[46] Koroleva I.A., Nazarenko M.S., Kucher A.N. (2017). Role of microRNA in Development of Instability of Atherosclerotic Plaques. Biochemistry (Moscow).

[47] Vishnoi A., Rani S. (2017). MiRNA Biogenesis and Regulation of Diseases: An Overview. Methods Mol. Biol..

[48] Lucherini O.M., Obici L., Ferracin M., Fulci V., McDermott M.F., Merlini G., Muscari I., Magnotti F., Dickie L.J., Galeazzi M. (2013). First report of circulating microRNAs in tumour necrosis factor receptor-associated periodic syndrome (TRAPS). PLoS ONE.

[49] Harrison S.R., Scambler T., Oubussad L., Wong C., Wittmann M., McDermott M.F., Savic S. (2018). Inositol-Requiring Enzyme 1-Mediated Downregulation of MicroRNA (miR)-146a and miR-155 in Primary Dermal Fibroblasts across Three TNFRSF1A Mutations Results in Hyperresponsiveness to Lipopolysaccharide. Front. Immunol..

[50] Bluher M. (2014). Adipokines—Removing road blocks to obesity and diabetes therapy. Mol. Metab..

[51] Cantarini L., Obici L., Simonini G., Cimaz R., Bacarelli M.R., Merlini G., Vitale A., Lucherini O.M., Brizi M.G., Galeazzi M. (2012). Serum leptin, resistin, visfatin and adiponectin levels in tumor necrosis factor receptor-associated periodic syndrome (TRAPS). Clin. Exp. Rheumatol..

[52] Li L., Yang G., Shi S., Yang M., Liu H., Boden G. (2009). The adipose triglyceride lipase, adiponectin and visfatin are downregulated by tumor necrosis factor-alpha (TNF-alpha) in vivo. Cytokine.

[53] La Cava A., Matarese G. (2004). The weight of leptin in immunity. Nat. Rev. Immunol..

[54] Pucino V., Lucherini O.M., Perna F., Obici L., Merlini G., Cattalini M., La Torre F., Maggio M.C., Lepore M.T., Magnotti F. (2016). Differential impact of high and low penetrance TNFRSF1A gene mutations on conventional and regulatory CD4+ T cell functions in TNFR1-associated periodic syndrome. J. Leukoc. Biol..

[55] Todd I., Radford P.M., Daffa N., Bainbridge S.E., Powell R.J., Tighe P.J. (2007). Mutant tumor necrosis factor receptor associated with tumor necrosis factor receptor-associated periodic syndrome is altered antigenically and is retained within patients’ leukocytes. Arthritis Rheum..

[56] Aganna E., Hammond L., Hawkins P.N., Aldea A., McKee S.A., van Amstel H.K., Mischung C., Kusuhara K., Saulsbury F.T., Lachmann H.J. (2003). Heterogeneity among patients with tumor necrosis factor receptor-associated periodic syndrome phenotypes. Arthritis Rheum..

[57] Hull K.M., Drewe E., Aksentijevich I., Singh H.K., Wong K., McDermott E.M., Dean J., Powell R.J., Kastner D.L. (2002). The TNF receptor-associated periodic syndrome (TRAPS): Emerging concepts of an autoinflammatory disorder. Medicine.

[58] Lachmann H.J. (2017). Periodic fever syndromes. Best Pr. Res. Clin. Rheumatol..

[59] Pettersson T., Kantonen J., Matikainen S., Repo H. (2012). Setting up TRAPS. Ann. Med..

[60] Rezaei N. (2006). TNF-receptor-associated periodic syndrome (TRAPS): An autosomal dominant multisystem disorder. Clin. Rheumatol..

[61] Nezos A., Argyropoulou O.D., Klinaki E., Marketos N., Karagianni P., Eliopoulos E., Vlachoyiannopoulos P., Maritsi D.N., Tzioufas A.G. (2019). Molecular and clinical spectrum of four pedigrees of TRAPS in Greece: Results from a national referral center. Rheumatology.

[62] Zhao M., Luo Y., Wu D., Yang Y., Sun Y., Wang R., Shen M. (2019). Clinical and genetic features of Chinese adult patients with tumour necrosis factor receptor-associated periodic fever syndrome. Rheumatology.

[63] Jesus A.A., Fujihira E., Watase M., Terreri M.T., Hilario M.O., Carneiro-Sampaio M., Len C.A., Oliveira S.K., Rodrigues M.C., Pereira R.M. (2012). Hereditary autoinflammatory syndromes: A Brazilian multicenter study. J. Clin. Immunol..

[64] Cantarini L., Lucherini O.M., Cimaz R., Baldari C.T., Bellisai F., Rossi Paccani S., Laghi Pasini F., Capecchi P.L., Sebastiani G.D., Galeazzi M. (2009). Idiopathic recurrent pericarditis refractory to colchicine treatment can reveal tumor necrosis factor receptor-associated periodic syndrome. Int. J. Immunopathol. Pharm..

[65] Cantarini L., Lucherini O.M., Vitale A., Sabadini L., Brizi M.G., Frediani B., Muscari I., Galeazzi M. (2013). Expanding spectrum of TNFRSF1A gene mutations among patients with idiopathic recurrent acute pericarditis. Intern. Med. J..

[66] Rigante D., Cantarini L., Imazio M., Lucherini O.M., Sacco E., Galeazzi M., Brizi M.G., Brucato A. (2011). Autoinflammatory diseases and cardiovascular manifestations. Ann. Med..

[67] Schmaltz R., Vogt T., Reichrath J. (2010). Skin manifestations in tumor necrosis factor receptor-associated periodic syndrome (TRAPS). Dermato Endocrinol..

[68] Toro J.R., Aksentijevich I., Hull K., Dean J., Kastner D.L. (2000). Tumor necrosis factor receptor-associated periodic syndrome: A novel syndrome with cutaneous manifestations. Arch. Derm..

[69] Rigante D., Capoluongo E. (2011). The plodding diagnosis of monogenic autoinflammatory diseases in childhood: From the clinical scenery to laboratory investigation. Clin. Chem. Lab. Med..

[70] Piram M., Frenkel J., Gattorno M., Ozen S., Lachmann H.J., Goldbach-Mansky R., Hentgen V., Neven B., Stojanovic K.S., Simon A. (2011). A preliminary score for the assessment of disease activity in hereditary recurrent fevers: Results from the AIDAI (Auto-Inflammatory Diseases Activity Index) Consensus Conference. Ann. Rheum. Dis..

[71] Federici S., Sormani M.P., Ozen S., Lachmann H.J., Amaryan G., Woo P., Kone-Paut I., Dewarrat N., Cantarini L., Insalaco A. (2015). Evidence-based provisional clinical classification criteria for autoinflammatory periodic fevers. Ann. Rheum. Dis..

[72] Gattorno M., Hofer M., Federici S., Vanoni F., Bovis F., Aksentijevich I., Anton J., Arostegui J.I., Barron K., Ben-Cherit E. (2019). Classification criteria for autoinflammatory recurrent fevers. Ann. Rheum. Dis..

[73] Arostegui J.I., Solis P., Aldea A., Cantero T., Rius J., Bahillo P., Plaza S., Vives J., Gomez S., Yague J. (2005). Etanercept plus colchicine treatment in a child with tumour necrosis factor receptor-associated periodic syndrome abolishes auto-inflammatory episodes without normalising the subclinical acute phase response. Eur. J. Pediatr..

[74] Cantarini L., Rigante D., Lucherini O.M., Cimaz R., Laghi Pasini F., Baldari C.T., Benucci M., Simonini G., Di Sabatino V., Brizi M.G. (2010). Role of etanercept in the treatment of tumor necrosis factor receptor-associated periodic syndrome: Personal experience and review of the literature. Int. J. Immunopathol. Pharm..

[75] Bulua A.C., Mogul D.B., Aksentijevich I., Singh H., He D.Y., Muenz L.R., Ward M.M., Yarboro C.H., Kastner D.L., Siegel R.M. (2012). Efficacy of etanercept in the tumor necrosis factor receptor-associated periodic syndrome: A prospective, open-label, dose-escalation study. Arthritis Rheum..

[76] Drewe E., McDermott E.M., Powell P.T., Isaacs J.D., Powell R.J. (2003). Prospective study of anti-tumour necrosis factor receptor superfamily 1B fusion protein, and case study of anti-tumour necrosis factor receptor superfamily 1A fusion protein, in tumour necrosis factor receptor associated periodic syndrome (TRAPS): Clinical and laboratory findings in a series of seven patients. Rheumatology.

[77] Stojanov S., Dejaco C., Lohse P., Huss K., Duftner C., Belohradsky B.H., Herold M., Schirmer M. (2008). Clinical and functional characterisation of a novel TNFRSF1A c.605T>A/V173D cleavage site mutation associated with tumour necrosis factor receptor-associated periodic fever syndrome (TRAPS), cardiovascular complications and excellent response to etanercept treatment. Ann. Rheum. Dis..

[78] Drewe E., Huggins M.L., Morgan A.G., Cassidy M.J., Powell R.J. (2004). Treatment of renal amyloidosis with etanercept in tumour necrosis factor receptor-associated periodic syndrome. Rheumatology.

[79] Ter Haar N., Lachmann H., Ozen S., Woo P., Uziel Y., Modesto C., Kone-Paut I., Cantarini L., Insalaco A., Neven B. (2013). Treatment of autoinflammatory diseases: Results from the Eurofever Registry and a literature review. Ann. Rheum. Dis..

[80] Nedjai B., Hitman G.A., Quillinan N., Coughlan R.J., Church L., McDermott M.F., Turner M.D. (2009). Proinflammatory action of the antiinflammatory drug infliximab in tumor necrosis factor receptor-associated periodic syndrome. Arthritis Rheum..

[81] Kaymakcalan Z., Sakorafas P., Bose S., Scesney S., Xiong L., Hanzatian D.K., Salfeld J., Sasso E.H. (2009). Comparisons of affinities, avidities, and complement activation of adalimumab, infliximab, and etanercept in binding to soluble and membrane tumor necrosis factor. Clin. Immunol..

[82] Furst D.E., Wallis R., Broder M., Beenhouwer D.O. (2006). Tumor necrosis factor antagonists: Different kinetics and/or mechanisms of action may explain differences in the risk for developing granulomatous infection. Semin. Arthritis Rheum..

[83] Akasbi N., Soyfoo M.S. (2015). Successful treatment of tumor necrosis factor receptor-associated periodic syndrome (TRAPS) with tocilizumab: A case report. Eur. J. Rheumatol..

[84] Hosoya T., Mizoguchi F., Hasegawa H., Miura K., Koike R., Kubota T., Miyasaka N., Kohsaka H. (2015). A Case Presenting with the Clinical Characteristics of Tumor Necrosis Factor (TNF) Receptor-associated Periodic Syndrome (TRAPS) without TNFRSF1A Mutations Successfully Treated with Tocilizumab. Intern. Med..

[85] Vaitla P.M., Radford P.M., Tighe P.J., Powell R.J., McDermott E.M., Todd I., Drewe E. (2011). Role of interleukin-6 in a patient with tumor necrosis factor receptor-associated periodic syndrome: Assessment of outcomes following treatment with the anti-interleukin-6 receptor monoclonal antibody tocilizumab. Arthritis Rheum..

[86] La Torre F., Muratore M., Vitale A., Moramarco F., Quarta L., Cantarini L. (2015). Canakinumab efficacy and long-term tocilizumab administration in tumor necrosis factor receptor-associated periodic syndrome (TRAPS). Rheumatol. Int..

[87] Lachmann H.J., Quartier P., So A., Hawkins P.N. (2011). The emerging role of interleukin-1beta in autoinflammatory diseases. Arthritis Rheum..

[88] Simon A., Bodar E.J., van der Hilst J.C., van der Meer J.W., Fiselier T.J., Cuppen M.P., Drenth J.P. (2004). Beneficial response to interleukin 1 receptor antagonist in traps. Am. J. Med..

[89] Gattorno M., Pelagatti M.A., Meini A., Obici L., Barcellona R., Federici S., Buoncompagni A., Plebani A., Merlini G., Martini A. (2008). Persistent efficacy of anakinra in patients with tumor necrosis factor receptor-associated periodic syndrome. Arthritis Rheum..

[90] Gentileschi S., Rigante D., Vitale A., Sota J., Frediani B., Galeazzi M., Cantarini L. (2017). Efficacy and safety of anakinra in tumor necrosis factor receptor-associated periodic syndrome (TRAPS) complicated by severe renal failure: A report after long-term follow-up and review of the literature. Clin. Rheumatol..

[91] Obici L., Meini A., Cattalini M., Chicca S., Galliani M., Donadei S., Plebani A., Merlini G. (2011). Favourable and sustained response to anakinra in tumour necrosis factor receptor-associated periodic syndrome (TRAPS) with or without AA amyloidosis. Ann. Rheum. Dis..

[92] Grimwood C., Despert V., Jeru I., Hentgen V. (2015). On-demand treatment with anakinra: A treatment option for selected TRAPS patients. Rheumatology.

[93] Brizi M.G., Galeazzi M., Lucherini O.M., Cantarini L., Cimaz R. (2012). Successful treatment of tumor necrosis factor receptor-associated periodic syndrome with canakinumab. Ann. Intern. Med..

[94] Lopalco G., Rigante D., Vitale A., Frediani B., Iannone F., Cantarini L. (2015). Tumor necrosis factor receptor-associated periodic syndrome managed with the couple canakinumab-alendronate. Clin. Rheumatol..

[95] Gattorno M., Obici L., Cattalini M., Tormey V., Abrams K., Davis N., Speziale A., Bhansali S.G., Martini A., Lachmann H.J. (2017). Canakinumab treatment for patients with active recurrent or chronic TNF receptor-associated periodic syndrome (TRAPS): An open-label, phase II study. Ann. Rheum. Dis..

[96] De Benedetti F., Gattorno M., Anton J., Ben-Chetrit E., Frenkel J., Hoffman H.M., Kone-Paut I., Lachmann H.J., Ozen S., Simon A. (2018). Canakinumab for the Treatment of Autoinflammatory Recurrent Fever Syndromes. N. Engl. J. Med..

